# Herbal Medicines for Asthmatic Inflammation: From Basic Researches to Clinical Applications

**DOI:** 10.1155/2016/6943135

**Published:** 2016-07-20

**Authors:** Fang Liu, Nan-Xia Xuan, Song-Min Ying, Wen Li, Zhi-Hua Chen, Hua-Hao Shen

**Affiliations:** ^1^Department of Respiratory and Critical Care Medicine, Second Affiliated Hospital, Zhejiang University School of Medicine, Hangzhou 310009, China; ^2^State Key Laboratory of Respiratory Diseases, Guangzhou 510120, China

## Abstract

Asthma is one of the most common chronic inflammatory disorders, associated with reversible airflow obstruction, airway hyperresponsiveness, and airway remodeling. This disease has a significant impact on individuals, their families, and society. Standardized therapeutics such as inhaled corticosteroid in combination with long acting *β*2 agonist have been applied for asthma control; however, complementary and alternative medicines, especially herbal medicines, are still widely used all over the world. A growing body of literature suggests that various herbals or related products might be effective in inhibiting asthmatic inflammation. In this review, we summarize recent advances about the mechanistic studies of herbal medicines on allergic airway inflammation in animal models and their potential application into clinic for asthma control.

## 1. Introduction

Asthma is a chronic inflammatory disease characterized by reversible airway obstruction, airway hyperresponsiveness (AHR), infiltration of inflammatory cells, mucus hypersecretion, and airway remodeling [1]. It affects 300 million individuals worldwide, with the prevalence ranging from 1% to 18% of the population in different countries [2]. A variety of immune cells, structure cells in the lung, cytokines, chemokines, adhesion molecules, and signaling pathways contributes to the asthmatic pathogenesis. Although standardized therapeutics such as inhaled corticosteroid (ICS) in combination with long acting *β*2 agonist (LABA) have been used to control asthma symptoms, complementary and alternative medicine (CAM) is still common all over the world. A survey involved 7685 individuals aged 55 or older with current asthma was performed recently in USA, and it showed that CAM use in the older adult asthmatic population was frequent, with nearly 40% using some type of CAM [3]. Another survey about traditional Chinese medicine (TCM) for pediatric asthma in Taiwan showed that 57.95% (*N* = 26 585) of the investigated children had used TCM [4].

Based on the facts that TCM or CAM is widely used in asthma control, increasing basic or clinical studies have been conducted to investigate the molecular mechanisms or clinical applications of herbal medicines for asthma therapy. Given the fact that asthma pathogenesis is complex, the roles and effective targets of these herbal products in asthma therapy are also very complicated.

In general, basic researches are all trying to separate effective monomer from herbs or herbal formula for asthma study, while most clinical researches still only focus on the efficacy for patients using an intact traditional formula. We will illuminate the major achievements of them, respectively, in this review.

## 2. Basic Researches

Basic researches on herbal asthma therapy can be summarized into nine aspects according to the mechanism summarized below. Those researches that only reported some T helper 1 (Th1) cell or T helper 2 (Th2) cell cytokines, for example, Interleukin-4 (IL-4), IL-5, IL-13, and interferon-*γ* (IFN-*γ*), altered after herbs intervention without any further mechanistic studies will not be expatiated in our paper, as they can be affected by many factors.

### 2.1. Targeting the Th1/Th2 Imbalance

It is generally accepted that Th1/Th2 imbalance is responsible for the development of allergic asthma. Th1 cells secrete IFN-*γ*, IL-12, and tumor necrosis factor-*β* (TNF-*β*), whereas Th2 cells secrete IL-4, IL-5, and IL-13 [5]. IL-4 together with IL-13 causes isotype class-switching of B cells towards Immunoglobulin-E (IgE) synthesis, which can bind to high-affinity receptors on mast cells and basophils, and leads to subsequent activation of these cells. IL-5 activates eosinophils and attracts them to the lung, where they secrete numerous inflammatory cytokines and chemokines. IL-13 also directly affects the airway epithelium, including increases in goblet cell differentiation, activation of fibroblasts, and bronchial hyperresponsiveness [5–7]. These cytokines may also in turn affect the Th1/Th2 balance [5, 8]. It has been acknowledged that two transcription factors, that is, T-bet and GATA-3, are responsible for Th1/Th2 balance. Further, GATA-3 and T-bet can be influenced by IL-12, IFN-*γ*, or IL-4 via the signal transducers and activators of transcription (STAT), that is, STAT4, STAT1, and STAT6, respectively [5].

Extractives from* Astragalus*,* Panax ginseng*,* Saururus chinensis*,* Psoralea*, and* Ligustrazine* [9] were reported a similar mechanism of decreasing the ratio of GATA3/T-bet expression level. Typically, Jin et al. [10] investigated the effects of the boiling water extract of* Psoralea fructus* (PF) and psoralen, an active ingredient of PF, on Th2 clone (D10.G.4.1) cells* in vitro* and* in vivo*, and interpreted their effect as suppressing GATA-3 protein expression. Similarly, Chen et al. [11] found that a single compound, Bavachinin, isolated from PF decreased the GATA-3 function by reducing the stability of GATA-3 mRNA and further suggested that Bavachinin may suppress the binding or coactivating function but not expression of pSTAT6. In their further study [12], two new derivatives of Bavachinin with a better water solubility were investigated, and one of these two derivatives not only inhibited GATA-3 mRNA production but also increased T-bet mRNA production. However, clinical research for* Psoralea fructus* in treating asthma is lacking. Only few case reports [13] involving* Psoralea fructus* related recipe in Chinese can be found but provided limited evidence.

Efficacy on STAT6 was reported in extracts from* Scutellaria baicalensis* [14] and* Cnidii monnieri* [15]. Chiu et al. [15] explored the effects of Osthol (*Cnidii monnieri fructus* extract) on epithelial cells using human bronchial epithelial cells (BEAS-2B)* in vitro*. Their research demonstrated that Osthol suppressed IL-4-induced eotaxin (a key mediator in allergic diseases with eosinophilic infiltration) in epithelial cells via inhibition of STAT6 expression.

Besides, without further mechanism revealed, efficacy on Th1/Th2 cytokines was also observed in taraxasterol (isolated from* Taraxacum officinale*) [16],* Duchesnea chrysantha* ethanol extract [17],* Echinacea purpurea* complex [18], matrine (isolated from* Sophora flavescens*) [19],* Zingiber officinale* (both ethanol and aqueous extracts) [20],* Actinidia polygama* fructus extract [21], and formulas like* Sam So Eum* [22] and* Qu Feng Xuan Bi* [23].

### 2.2. Effect on MAPK and NF-*κ*B Signaling Pathways

Mitogen-activated protein kinases (MAPKs), which comprise three major subgroups, that is, extracellular signal-related kinase 1/2 (ERK1/2), p38, and c-Jun N-terminal kinase 1/2 (JNK1/2), play critical roles in the activation of inflammatory cells [24]. Nuclear factor kappa B (NF-*κ*B) is an important transcription factor involved in the expression of various proinflammatory genes. Increased activation of NF-*κ*B has been observed in the lungs after allergen challenge and in airway epithelial cells and macrophages from asthmatic patients [25]. Many studies have reported that allergic asthma could be improved by regulating the activation of MAPK and NF-*κ*B signaling pathways [26, 27].

Herbs that were extracted targeting on MAPKs include* Scutellaria baicalensis* [28, 29],* Panax ginseng* [30],* Saururus chinensis* [31],* Artemisia annua* [32],* Magnoliae flos* [33, 34], and* Crocus sativus* [35]. While NF-*κ*B inhibitors can be found in* Scutellaria baicalensis* [36],* Astragalus* [37, 38],* Saururus chinensis* [31],* Astilbe chinensis* [39],* Artemisia annua* [32], and* Garlic* [40]. Although they performed similar mechanisms, minor differences could be noticed. Some extracts like dihydroartemisinin [32] (isolated from* Artemisia annua*) and meso-Dihydroguaiaretic acid [31] (isolated from* Saururus chinensis*) act as inhibitors of MAPKs and NF-*κ*B meanwhile, whereas some extracts isolated from the same herb may inhibit either MAPKs or NF-*κ*B, respectively, for example, Oroxylin A [36] and Baicalin [28, 29] (both isolated from* Scutellaria baicalensis*). Details can be found in Table 1.

### 2.3. Targeting the Treg/Th17 Cells

T-regulatory cells (Tregs) are a heterogeneous group of cells that play a central role in maintaining the homeostasis of pulmonary immunity by establishing immune tolerance to nonharmful antigens or suppressing effector T cell immunity. The specification of Treg subset is driven by transcription factor forkhead box P3 (Foxp3) [5, 75–78]. Th17 cells are key players in chronic lung inflammation, including asthma. Steroid-resistant asthma and neutrophil-mediated asthma have been proved to be related to Th17 cells. IL-17 also directly affects the airway smooth muscle by inducing allergen-induced airway hyperresponsiveness [79–82]. ROR*γ*t is found to be the transcription factor related to Th17, which is required to activate IL-17 production in Th17 cells [5]. Increased expressions of IL-17A and IL-17F have been shown in lung tissue of asthma patients [6]. Thus, herbal treatments targeting Foxp3 and ROR*γ*t have been revealed gradually.

Extracts from* Astragalus* [46, 48],* Panax ginseng* [52],* Crocus sativus* [60],* Ligustrazine* [9],* and Anoectochilus formosanus* [64] were observed increasing Tregs and enhancing Foxp3 mRNA expression. In particular, ligustrazine, isolated from* Ligustrazine* (*Chuan Qiong*), was reported modulating the expression of not only T-bet/Gata-3 but also Foxp3/ROR*γ*t [9]. Ji et al. [9] noticed that ligustrazine reduced not only eosinophils but neutrophils in the BALF of asthmatic mouse model, implying that it could have potential for use in alleviation of neutrophilic asthma.

Besides, there were more studies about herbal treatments in other Th17-related inflammatory diseases, such as* Andrographis paniculata*,* Scutellaria baicalensis*,* Tripterygium wilfordii, and Wedelia chinensis* [33, 83–85]. However, whether these herbs work similarly in asthma needs further investigations.

### 2.4. Effect on Lung Dendritic Cells (DCs)

Dendritic Cells (DCs) participated not only in the differentiation of T helper cells but also in IL-12 production and CD8+ T cell stimulation via antigen uptake. Two subsets of blood DCs, that is, myeloid and plasmacytoid DCs, were identified based on the expression of CD11c [5]. Most CD11c+ myeloid DCs in the lung are immature, which express relatively low levels of major histocompatibility complex (MHC) class II, and have a high capacity of antigen uptake but poor T cell stimulating activity [5]. Thus, inhibiting functional differentiation of pulmonary immature DC to mature DC may be a strategy to restrict the activation of T cells.

Lee et al. [63] discussed the effect of* Artemisia iwayomogi* polysaccharide-1 (AIP1) on DC functions. They observed significantly reduced levels of MHC II in DCs of the AIP1 treated group, suggesting that AIP1 could reduce the expression of MHC II molecules on pulmonary DC. They also reported that AIP1 diminished the allergenic T cell stimulating ability of DCs derived from bone marrow in another study. These data suggested that AIP1 could inhibit functional differentiation of pulmonary DCs* in vivo*.

### 2.5. Effect on Mast Cell Degranulation

Mast cell degranulation, which can be triggered by antigen-mediated cross-linking of IgE bound to Fc*ε*R1 surface receptors or changes in the surrounding local tissue environment, plays an important role in asthmatic response. As a result, many of the mediators that are stored or newly synthesized by the mast cells are released attracting leukocytes (eosinophils, basophils, Th2 lymphocytes, and neutrophils) to the inflammatory site and amplify the inflammatory response [86]. Hence, inhibiting mast cell degranulation will be helpful for asthma treating.

We gathered three extracts that associated with this process: Oroxylin A [43], Bakkenolide B [66], and Petatewalide B [67]. The first one is isolated from* Scutellaria baicalensis* while the next two are from* Petasites japonicus*. It is worth mentioning that both Bakkenolide B and Petatewalide B do not inhibit antigen-induced Ca2+ increases in mast cells, which suggested that Bakkenolide B/Petatewalide B induced inhibition of degranulation seems not to be mediated via the inhibition of Ca2+ channel or Ca2+ increase in mast cells. As for Oroxylin A, no detailed mechanisms were provided to expand the phenomenon of inhibiting mast cell degranulation. Further mechanistic investigations on these extracts are necessary.

### 2.6. Effect on Oxidative Stress

Oxidative stress plays an important role in the pathogenesis of most airway diseases, particularly when inflammation is prominent. Recently, heme oxygenase-1 (HO-1) was shown to be induced in the airways of patients with asthma. As a natural antioxidant defense, HO-1 exerts cytoprotective reactions against oxidative cell injury. Greater HO-1 expression may mitigate asthma symptoms and suppressed IL-13-induced goblet cell hyperplasia and MUC5AC production [87–89]. Hence, targeting on HO-1 or its transcription factor nuclear factor E2-related factor 2 (Nrf-2) [90] is a considerable strategy for asthma control.

To date, a variety of HO-1 activator can be extracted from* Saururus chinensis* [54, 55],* Phytolacca esculenta* [68],* Garlic* [40], and* soshiho-tang* [73]. Among them, diallyl-disulfide (isolated from* Garlic*) [40] and esculentoside A (isolated from* Phytolacca esculenta*) [68] have been further proved as Nrf-2 activators. Beyond these, artesunate from* Artemisia annua* was also observed suppressing prooxidants and restoring expression of antioxidants via activation of Nrf-2 [62].

There are also some studies that only showed a reduced level of oxidative stress marker reactive oxygen species (ROS) when extracts like ethanol extracts of* Mentha* [59] and* Petasites japonicus* [65] intervened. The mechanisms by which they reduce ROS level need further study.

### 2.7. Effect on Relaxing Airway Smooth Muscle

Airway contraction is an important feature of asthma, and recent strategies to relax airway smooth muscle include antihistamine and anticholinergic and *β*2-adrenoreceptors stimulation and Ca2+ signaling blocking [61].

Mokhtari-Zaer et al. [61] summarized the* Crocus sativus*'s (saffron's) effect on relaxing airway smooth muscle in a review. Aqueous-ethanolic extract of* Crocus sativus* and safranal were mentioned in their article and showed multiple effects including antihistamine, anticholinergic, and *β*2-adrenoreceptors stimulation according to four published studies.

Yang et al. [69] identified that trifolirhizin, a flavonoid compound isolated from* Sophora flavescens*, was responsible for inhibiting acetylcholine induced airway smooth muscle (ASM) contraction independent of *β*2-adrenoceptors.

Aqueous methanolic extract from* Zingiber officinale* (ginger) was also reported having effect on acetylcholine induced airway contraction. Ghayur et al. [70] indicated that its effects were associated with Ca2+ signaling, possibly via blocking Ca2+ channels on plasma membrane.

### 2.8. Effect on Airway Remodeling

It is believed that airway smooth muscle (ASM) cell proliferation and migration play important roles in airway remodeling. Both platelet-derived growth factor (PDGF) and transforming growth factor-*β* (TGF-*β*) are reported to be related to airway remodeling [91]. Recently, TGF-*β*1/Smad signal pathway was found to be one of the important mechanisms for signal conduction in asthma airway remodeling [92].

Astragaloside IV and Skullcapflavone II, extracts from* Astragalus* and* Scutellaria baicalensis,* respectively, were reported to attenuate the allergen-induced airway remodeling in mice, likely through inhibition of TGF-*β*1 [37, 41]. Further research was performed by Jang et al. [41] and indicated that Skullcapflavone II elevated Smad7 and suppressed Smad2/3 expression, which was responsible for TGF-*β*1 inhibition. As for* Astragalus,* our group researched several formulas using* Astragalus* as key component acting on TGF-*β*1/Smad signal pathway. The one* Astragali-Cordyceps* Mixtura [50] decreased TGF-*β*1 expression and recovered Smad7 protein expression, and the other one* Astragali radix Antiasthmatic Decoction* (AAD) [93] was also found to improve the symptoms of allergic airway remodeling through inhibition of Th2 cytokines and TGF-*β*1. In a recent study, we also noticed that Suhuang antitussive capsule, a traditional Chinese medication, significantly attenuated the allergen-induced AHR, inflammation, and remodeling in mice, likely through inhibition of IL-13 and TGF-*β*1 [94].

### 2.9. Effect on the Arachidonic Acid Metabolism Pathway (AAMP)

Arachidonic acid (AA) from the diet or after synthesis is stored in membrane phospholipids and is liberated under appropriate stimulatory conditions by the enzyme phospholipase A2 (PLA2). Arachidonic acid is then metabolized by three main classes of enzymes (cyclooxygenases (COX), lipoxygenases (LOX), and p450 epoxygenases) and all products of these three pathways like prostaglandin E2 (PGE2), prostaglandin D2 (PGD2), leukotrienes (LTs), and so forth are related to inflammatory and anaphylactic reaction. To be specific, PGE2 has been thought of as a potent proinflammatory mediator and has many beneficial functions in the lung tissues, such as the inhibition of inflammatory cell recruitment, reduction of leukotrienes and PGD2, and decrease of Th2 differentiation, thereby modulating inflammation and tissue repair. LTs (LTB4, LTC4, LTD4, etc.) are also thought to be important mediators of airway inflammation and airway obstruction in asthma. LTB4 can act as a neutrophil chemoattractant [53, 95, 96]. Thus, strategies targeting AA metabolism are effective in many inflammatory diseases.

Extracts from* Scutellaria baicalensis* [42],* Panax ginseng* [53],* Saururus chinensis* [57],* Sceptridium ternatum* [58],* Aralia cordata* [71], and eucalyptol (1.8-cineole) [74] have been found targeting different steps of the arachidonic acid metabolism pathway (AAMP). Details can be checked in Table 1.

Particularly, there are 4 herbs we would like to review individually as follows for their multiple function of antiasthma and popularity in basic researches.

*Scutellaria baicalensis* is a multifunctional traditional herb. Its extracts include Skullcapflavone II, Baicalein, Oroxylin A, wogonin, and Baicalin, which showed different but maybe cooperative functions in treating asthma. All of them have been proved in animal experiment or* in vitro* but not in human as mentioned before. And their results only showed simplex function on some cytokines or genes related to asthma. Whether they can benefit the whole interaction when asthma occurred is still unclear. For example, Jang et al.'s experiment [41] indicated Skullcapflavone II's function on TGF-*β*1/Smad signaling pathways. They observed a decreased level of TGF-*β*1 in BALF, elevated Smad7 expression, and suppressed Smad2/3. However, as a pleiotropic and multifunctional growth factor, TGF-*β*1 also exerts immunosuppressive effects on asthma progression, and therapies targeting on TGF-*β*1 are still controversial, although it has been expatiated that TGF-*β*1 is responsible for airway remodeling [97].

Another multifunctional herb with relative sufficient studies is* Astragalus membranaceus.* We have showed above that its extract has been reported acting on GATA3/T-bet and TGF-*β*1/Smad and NF-*κ*B signal pathway and Tregs within last few years. Among them, Astragaloside IV should be underlined here for the fact that it nearly performed all effects that* Astragalus membranaceus* possessed for treating asthma. However, it is a pity that although abundant studies were performed on Astragaloside IV, no clinical research on it is available to date.

Extracts (RG-II, CVT-E002, and ginsan) from* Panax* were also reported acting on different pathways involving GATA3/T-bet, MAPK, and Tregs and arachidonic acid metabolism pathway in animal models or* in vitro*. But whether these results can be applied to human is undetermined. It is worth mentioning that CVT-E002 has been well proved to reduce respiratory infection in patients with chronic lymphocytic leukemia and prevent acute respiratory illness in institutionalized older adults [98, 99]. It may partly reflect its immunoregulation function on human. Researches on its efficacy on asthma patients are desired.

*Saururus chinensis* (SC) is an effective antioxidant. Three of its extracts (saucerneol D [54], a subfraction of its ethanol extract, and sauchinone [55]) showed similar effect of antioxidant through upregulating the expression of HO-1. Among them sauchinone also suppressed GATA-3 activity [56]. Recently, a novel extract of SC named meso-Dihydroguaiaretic acid was expounded by Song and his colleagues [31]. It exhibited a protective effect on allergic airway inflammation through inhibiting the Th2 inflammation attributed to its inhibition of the NF-*κ*B and MAPK. Besides, its ethanol extract's action on arachidonic acid metabolism pathway was reported in this year [57]. Antiasthmatic effect for SC extracts is drawing wide attention in the last few years.

## 3. Clinical Studies

The clinical research of herbal therapy application in asthma is limited. Three meta-analysis or systematic reviews were published successively in 2007, 2008, and 2010 [100–102]. However, all of them could not get sufficient evidence to make recommendations for herbal treatment for asthma after comprehensive analysis of the efficacy and safety. Arnold et al. [100] evaluated effects of herbal medicines on lung function, reduction in use of corticosteroids, symptom scores, physical sign scores, use of reliever medications, health related quality of life, and adverse effects comparing with placebo, involving 21 different herbs or herbal formulas. Although a few of them had some effects on relief of symptoms, only boswellic acids (isolated from* Boswellia*) were reported to exert a relatively comprehensive effect on lung function, while the effects of other herbs were limited or inexact. In Clark et al.'s study [102], Mai-Men-Dong-Tang, Pycnogenol, Jia-Wei-Si-Jun-Zi-Tang, and* Tylophora indica* also showed potential to improve lung function. Moreover, 1.8-cineol (eucalyptol) was observed to reduce the use of corticosteroids and corticosteroid reduction tolerance (<7.5 mg) in both of their studies [100, 102].

In the last five years, some new clinical trials on herbal treatment emerged but most of them still focus on the efficacy for patients using an intact traditional or modified formula. The application of monomer extracts in clinic usually acts as adjuvant of standard asthma therapy according to the recent studies. In a noncomparative, multicenter trial [103], 148 patients with mild asthma taking ICS received NDC-052 (an extract from* Magnoliae flos*) for eight weeks. Their results showed that add-on NDC-052 besides ICS therapy had benefits in both ΔPEFR and asthma symptoms. Last year, a review by Ammon [104] showed multiple effects of* Boswellia serrata* extracts on immune system modulation in basic research, including inhibiting activation of NF*κ*B, mast cell stabilisation, and antioxidant and inhibitory action on 5-LOX. However, related clinical research in the past five years can only be found for reducing the need for inhalation therapy with ICS + LABAs [105]. Although its significant effect on lung function had been analyzed by Arnold et al. as mentioned before [100], further research is still lacking.

Regarding intact traditional or modified formula for asthma reported recently, there are three types of research interests in the past five years.

First, herbal formula is singly used to relieve asthmatic symptom. These studies paid attention to the syndrome scores and frequency of asthma acute attack although these formulas may have limited efficacy on asthma process or lung function improvement. Geng et al. [106] performed a randomized, single-blind, placebo controlled trial (sample size = 60) on intermittent asthmatic children aged 2 to 5 years. Their modified formula contained* Radix Astragali Mongolici* 10 g,* Rhizoma Polygonati Odorati* 10 g,* Fructus Ligustri Lucidi* 10 g,* Fructus Psoraleae* 10 g,* Radix Pseudostellariae* 3 g,* Fructus Schisandrae Chinensis* 3 g,* Fructus Jujubae* 10 g,* Concha Ostreae* 10 g, and* Endoconcha Sepiellae* 10 g. Their results showed that the formula reduced the number of intermittent asthma attacks, decreased the syndrome scores, and reduced the airway resistance in the children. Homogeneously, another formula, “Zhisou Powder,” is also observed to decrease the syndrome score and cough score of cough variant asthma, but it has no effect on airway responsiveness [107].

Second, maybe the most popular direction is usage of herbal formula as add-on therapy of standard medication to relieve asthmatic symptom and recurrence rate or strengthen the effect of standard medication. A randomized controlled research on 143 patients with moderate-severe asthma was performed by Tang and colleagues [108]. They applied their formula (containing 21 herbs and excipients) as add-on therapy of standard medication and showed decrease of exacerbation frequency and improvement of related syndrome scores, for example, asthma control test (ACT) score. Lin et al. [109] studied the effect of* Astragalus* plus hormone treatment in 90 asthmatic children. They showed that the effective rate of the* Astragalus* plus hormone group is significantly higher compared to using* Astragalus* or hormone only. The levels of PEFR and IFN-*γ* significantly increased and IL-4 obviously decreased in their effective cases. Besides, in another study, benefit of “chaipo granule” combined with routine treatment on refractory asthma was also discovered. “Chaipo granule” can act as a synergist of routine treatment according to their results [110]. Similar strengthening effect was also reported in the formula “Yupingfeng powder” [111].

Third, study in this research interests is according to a traditional administration method in China. Chinese called it “Jiu” (moxibustion), which uses herbs burning at the related acupoint of patients. Peoples today modified the method and are using percutaneous absorption herbal patch to treat asthma. Typically, Chen et al. [112] compared their modified percutaneous absorption herbal patch with salmeterol fluticasone inhalation for asthma of paracmasis. Their results showed a significant improvement in clinical symptom scores.

## 4. Conclusion Remarks

After all the above, it can be noticed that herbal therapy mainly applied to mild asthma or acted as adjuvant of standard asthma therapy in clinical researches. It seems to become a trend. It might be a good way to use complementary and alternative medicine (CAM) to help control symptom and reduce the drug dose for those patients receiving standard medication, as it has been proved by several researches [100–105, 108]. However, compared with the prosperous basic researches, clinical researches on asthma herbal therapy are relatively poor. It may be because many herbal extracts targeting on one or two factors of asthma may not be that meaningful when applied in clinic, as asthma is a disease with complex and multiple mechanisms. On the other hand, as the herbs were usually administrated as formula, simultaneously using extracts from different herbs with different mechanisms can also be a good research direction. It can be expected that they act synergetically and perform significant benefit on asthma when used simultaneously. Administrating purified extracts synergetically will have more precision than using intact formula directly.

Despite the remarkable achievements about herbal treatment for asthma during the past several years, several points should be addressed or be kept in mind in future studies. (1) Quality control of herbal medicines is always problematic, due to lack of standard procedures to make herb extractions, decoctions, or formula. Herbal patent drugs are generally of better quality, but there are few ones available for asthma researches. (2) Apparently, there is an urgent need for translational studies to transfer the current achievements on herbal medicines from animal works to clinical trials and finally to develop new clinical therapy. Current clinical trials for herbal medicines are limited, and thus more and large scaled, multicentre ones are extremely needed. Fortunately, well-designed and well-performed clinical trials for herbal medicines were appreciated by world-class journals [113], encouraging similar studies in asthma. (3) For clinical studies, the usage of herbal medicines for stable asthma is recommended. Asthma research in animal models usually cannot mimic the distinction between stable asthma and exacerbation. Herbal medicines might help to control stable asthma, while they may have limited effects during asthma exacerbations, since the herbs generally take longer time to exert therapeutic effects. (4) Clinical studies of herbal medicines in asthma control should target two major different goals. One is to take herbal medicines as a sole asthma control strategy, and the other is to use them as an add-on therapy for standard strategies, that is, to enhance the efficacy of or to reduce the usage of ICS. (5) Most of the current researches focused only on the eosinophilic phenotype of asthma, and few researches addressed other phenotypes like neutrophilic asthma, as the latter is usually severe asthma and is hard to control by current approaches. Thus, it is encouraged to conduct studies, both basic and clinical, to investigate the possible roles of herbal medicines in control of severe or neutrophilic asthma.

Nevertheless, herbal medicines for asthma hold out a cheerful prospect, which will eventually help to reduce the morbidity and mortality and increase the control levels of asthma worldwide.

## Figures and Tables

**Table 1:** Details of basic researches included in the paper.

Herbs	Material	Extracts	Asthmatic model	Route	Effective dose	Mechanism	
						Category	Details
*Scutellaria baicalensis*	Root	Skullcapflavone II [41]	OVA-induced Balb/c mice	Oral	10, 30 mg/kg/d *∗* 7 d	TGF-*β*1/Smad	Reduced TGF-*β*1 in BLAF, elevated Smad7, and suppressed Smad2/3 expression
	Root	Baicalein [42]	OVA-induced Balb/c mice	i.p.	10 mg/kg/d *∗* 6 d	AAMP	Reduced 12/15-LOX activity
	NA	Oroxylin A [36, 43]	OVA-induced Balb/c mice	Oral	15, 30, and 60 mg/kg/d *∗* 3 d	NF-*κ*B	Suppressed NF-*κ*B activation
			Specific IgE induced rat RBL-2H3 mast cells	*In vitro*	10, 30 *μ*M *∗* 30 min	Mast cells	Inhibited degranulation of mast cells
	Root	Wogonin [14]	OVA-induced Balb/c mice	Oral	10, 30 mg/kg/d *∗* 3 d	STAT6	Suppressed OVA-induced STAT6 activation
			IL-4 induced BEAS-2B cells	*In vitro*	10, 30, and 50 *μ*M*∗* 4 h	STAT6	Suppressed IL-4-induced eotaxin-3 expression via suppressing JAK1 and STAT6 activation
	Root	Baicalin [28, 29]	OVA-induced Balb/c mice	Oral	25, 50, and 100 mg/kg/d *∗* 4 w	MAPK	Inhibited RASM cell proliferation and migration by suppressing MAPK signal pathway
			Airway smooth muscle cells from SD rats (RASM)	*In vitro*	10, 25, and 100 nM *∗* 1 h		

*Astragalus*	NA	Aqueous extract [44]	OVA-induced C57BL/6 mice	Oral	3 *μ*g/kg/2 d *∗* 9 d	GATA3/T-bet	Decreased the ratio of the GATA3/T-bet mRNA levels
	Root	Astragaloside IV [37, 45–47]	OVA-induced Balb/c mice	Oral	50, 150 mg/kg/d *∗* 4 w	GATA3/T-bet	Decreased the ratio of the GATA3/T-bet expression level
			OVA-induced Balb/c mice	Oral	50, 150 mg/kg/d *∗* 4 w	—	Increased IFN-*γ* level
			OVA-induced Balb/c mice	Oral	20, 40 mg/kg/d *∗* 4 w	Foxp3/ROR*γ*t	Increased CD4+CD25+Foxp3+ Tregs and enhanced Foxp3 mRNA expression
			OVA-induced Balb/c mice	Oral	50 mg/kg/d *∗* 8 w	TGF-*β*1/Smad	(1) Reduced TGF-*β*1 expression
						NF-*κ*B	(2) Inhibited TSLP expression
	NA	Aqueous extract [48]	OVA-induced SD rats	Oral	5, 10 g/kg/d *∗* 4 w	Foxp3/ROR*γ*t	Increased CD4+CD25+Foxp3+ Tregs and enhanced Foxp3+ mRNA expression
	NA	Aqueous extract [49]	OVA-induced C57BL/6 mice	i.p.	10 g/kg/d *∗* 4 w	—	Increased Th1/Th2 cytokines' ratio
	NA	Formononetin & calycosin [38]	OVA-induced Balb/c mice	Oral	0.5 g/kg/2 d *∗* 4 w	NF-*κ*B	Suppressed NF-*κ*B activation

*Astragali-Cordyceps*	—	Astragali-Cordyceps suspensions [50]	OVA-induced C57BL/6 mice	Oral	6.5 g/kg/d *∗* 4 w	TGF-*β*1/Smad	Reduced TGF-*β*1 and elevated Smad7 expression in lung tissue

*Panax ginseng*	Root	Aqueous extract [30]	OVA-induced C57BL/6 mice	i.p.	20 mg/kg/d *∗* 3 d	MAPK	Inhibited CD40/CD40L ligation and MAPK signal pathway
	Leaves	Purified aqueous extract (RG-II) [51]	OVA-induced Balb/c mice	i.p.	20, 100 mg/kg/d *∗* 3 d	GATA3/T-bet	Decreased the ratio of the GATA3/T-bet expression level
	Root	Aqueous extract (CVT-E002) [52]	OVA-induced Balb/c mice	Oral	200 mg/kg/d *∗* 7 d	Foxp3/ROR*γ*t	Increased Tregs function and IL-10 level in BALF
	Root	Ginsan [53]	OVA-induced Balb/c mice	i.p.	100 mg/kg/2 d *∗* 4 w	AAMP	Upregulated COX-1 and COX-2 expression, leading to the increase of PGE2 in BALF

*Saururus chinensis*	Root	Saucerneol D [54]	OVA-induced Balb/c mice	Oral	20, 40 mg/kg/d *∗* 3 d	Antioxidant	Upregulated the expression of HO-1
	Aerial parts	A subfraction of ethanol extract [55]	RAW264.7 cells derived from BALB/c mice	*In vitro*	5, 50 *μ*g /mL *∗* 2 h	Antioxidant	Upregulated the expression of HO-1
	Aerial parts	Sauchinone [55, 56]	RAW264.8 cells derived from BALB/c mice	*In vitro*	2.5, 5, and 10 *μ*g /mL *∗* 2 h	Antioxidant	Upregulated the expression of HO-1
			OVA-induced Balb/c mice	i.p.	10, 100 mg/kg/2 d *∗* 5 d	GATA3/T-bet	Suppressed GATA-3 activity
	Root	Meso-Dihydroguaiaretic acid [31]	OVA-induced Balb/c mice	Oral	10, 30 mg/kg *∗* 2 w	NF-*κ*B & MAPK	Inhibited Th2 inflammation via inhibiting NF-*κ*B and MAPK
	Aerial parts	70% ethanol extract [57]	Bone marrow-derived mast cells from Balb/c mice	*In vitro*	0.8–50 *μ*g /mL *∗* 30 min	AAMP	Inhibited LTC4 and PGD2 level

*Sceptridium ternatum*	NA	70% ethanol extract [58]	OVA-induced Balb/c mice	Oral	2, 10, and 20 g/kg/d *∗* 10 d	—	Elevated the ratio of Th1/Th2
						AAMP	Decreased the cysLT1 mRNA levels

*Mentha*	Aerial parts	Ethanol extract [59]	OVA-induced Balb/c mice	Oral	100 mg/kg/d *∗* 6 d	—	(1) Inhibit increases in IgE, IL-4, and IL-5 in BALF and lung tissue
						Antioxidant	(2) Reduced the levels of ROS in BALF

*Psoralea*	Fructus	Aqueous extract [10]	OVA-induced Balb/c mice	Oral	10 g/kg/d *∗* 4 w	—	Inhibited the upregulation of IL-4 and IL-13 levels in BALF
	Fructus	Psoralen [10]	ConA stimulated D10.G4.1 cells	*In vitro*	0.08 mM *∗* 2 h	GATA3/T-bet	Suppressed the upregulation of IL-4, IL-5, IL-13, and GATA-3 protein expression
	Fructus	Bavachinin [11]	OVA-induced Balb/c mice	Oral	50 mg/kg/d *∗* 7 d	—	Suppressed IL-4, IL-5, and IL-13 in lung tissue and serum levels of IL-4, IgE
			ConA, IL-2, IL-4 stimulated 4GET mice spleen cells	*In vitro*	0.01 mM	GATA3/T-bet	Suppressed GATA-3 mRNA levels

*Astilbe chinensis*	NA	Astilbic acid [27]	OVA-induced Balb/c mice	Oral	30 mg/kg/d *∗* 3 d	NF-*κ*B	Suppressed NF-*κ*B activation

*Crocus sativus*	Flower	Crocetin [60]	OVA-induced C57BL/6 mice	Intranasal	3 *μ*g /d *∗* 1 w	Foxp3/ROR*γ*t	Increased Foxp3 through TIPE2 to activate Tregs
	Flower	Crocin [35]	OVA-induced Balb/c mice	Oral	100 mg/kg/d *∗* 5 d	MAPK	Inhibited the expression of lung eotaxin, p-ERK, p-JNK, and p-p38 level
	Flower	Safranal [61] (A review)	—	—	—	Relaxing ASM	Antihistamine and anticholinergic and *β*2-adrenoreceptors stimulation and Ca2+ signaling blocking

*Artemisia annua*	NA	Dihydroartemisinin [32]	OVA-induced Balb/c mice	Oral	30 mg/kg/d *∗* 3 d	NF-*κ*B & MAPK	Inhibited Th2 inflammation via inhibiting NF-*κ*B and MAPK
	NA	Artesunate [62]	OVA-induced Balb/c mice	i.p.	30 mg/kg/d	Antioxidant	Suppressed prooxidants and restoring expression of antioxidants via activation of Nrf-2

* Artemisia iwayomogi*	Leaves	Purified aqueous extract (AIP1) [63]	OVA-induced Balb/c mice	i.p.	5 mg/kg *∗* 6 times in 14 d	Dendritic cell	Reduced levels of MHC II in dendritic cells

*Ligustrazine *	NA	Ligustrazine [9]	OVA-induced C57BL/6 mice	i.p.	80 mg/kg/d *∗* 3 d	GATA3/T-bet	(1) Decreased the ratio of the GATA3/T-bet expression level
						Foxp3/ROR*γ*t	(2) Increased the ratio of Foxp3/ROR*γ*t

*Anoectochilus formosanus *	Whole	Aqueous extract [64]	OVA-induced Balb/c mice	NA	0.5, 1 g/kg/d *∗* 7 d	Foxp3/ROR*γ*t	Inhibited the decrease of Tregs in BALF

*Petasites japonicus*	NA	80% ethanol extract [65]	OVA-induced Balb/c mice	Oral	500 mg/kg/d *∗* 4 w	—	(1) Inhibit increases in OVA-specific IgE and IL-5 in BALF
						Antioxidant	(2) Reduced the levels of ROS in BALF
	Leaves	Bakkenolide B [66]	Rat RBL-2H3 mast cells & C57BL/6 mouse peritoneal macrophages	*In vitro*	1–10 *μ*g /mL *∗* 1 h	Mast cells	Inhibited degranulation in mast cells and suppressed iNOS in macrophages
	Leaves	Petatewalide B [67]		*In vitro*	10, 30 *μ*g /mL *∗* 1 h		

*Cnidii monnieri*	Fructus	Osthol [15]	IL-4/TNF-*α* induced BEAS-2B cells	*In vitro*	1–10 *μ*M *∗* 2 h	STAT6	Suppressed IL-4-induced eotaxin expression via suppressing STAT6 activation

*Magnoliae flos*	Leaves	Fargesin and epimagnolin [34]	A549 human alveolar epithelial cells	*In vitro*	3.1–100 *μ*g /mL	MAPK	Modulated NO synthesis via inhibiting ERK in human respiratory epithelial cells

*Phytolacca esculenta*	NA	Esculentoside A (EsA) [68]	OVA-induced Balb/c mice	i.p.	15 mg/kg/d *∗* 4 d	Antioxidant	Reduced the levels of ROS in BALF
			A549 human alveolar epithelial cells	*In vitro*	10, 20 mg/L *∗* 6 h	Antioxidant	Nrf-2 activator. Upregulated the expression of HO-1

*Garlic*	Oil	Diallyl-disulfide (DADS) [40]	OVA-induced Balb/c mice	Oral	30 mg/kg/d *∗* 3 d	Antioxidant	(1) Reduced the levels of ROS in BALF
						NF-*κ*B	(2) Suppressed NF-*κ*B activation
			RAW264.7 murine macrophage cell	*In vitro*	62.5–500 ng/mL *∗* 1 h	Antioxidant	Nrf-2 activator. Upregulated the expression of HO-1

*Sophora flavescens*	NA	Trifolirhizin [69]	Tracheal rings of OVA-induced Balb/c mice	*In vitro*	6 *μ*g /mL	Relaxing ASM	Inhibiting acetylcholine mediated ASM contraction

*Zingiber officinale*	Root	70% methanol extract [70]	Lung slices of Balb/c mice	*In vitro*	0.3, 1 mg/mL	Relaxing ASM	Inhibiting acetylcholine mediated ASM contraction via blocking Ca2+ channels

*Aralia cordata*	Root	7-Oxo-sandaracopimaric acid [71]	OVA-induced guinea pigs	Oral	25–100 mg/kg *∗* 3 in 24 h	AAMP	Inhibiting phospholipase A2 (PLA2) eosinophil peroxidase (EPO) activity in BALF

*Moringa oleifera *Lam.	Seed	*β*-Sitosterol [72]	OVA-induced guinea pigs	Oral	2.5 mg/kg/d *∗* 12 d	—	(1) Decreased the levels of TNF-*α*, IL-4, and IL-5 in BALF and serum
						Antihistamine	(2) Antihistamine

*Soshiho-tang*	—	Aqueous extract [73]	OVA-induced Balb/c mice	Oral	100, 200 mg/kg/d *∗* 6 d	Antioxidant	Upregulated the expression of HO-1

*NA*	NA	Eucalyptol (1.8-cineole) [74]	Monocytes from patients with asthma	*In vitro*	200 mg/d *∗* 3 d	AAMP	Inhibit LTB4 and PGE2

i.p. = intraperitoneal injection. AAMP = arachidonic acid metabolism pathway. TIPE2 = TNF-*α*-induced protein 8-like 2. NA = not available.
